# Uncovering the Links Between Dietary Sugar and Cancer: A Narrative Review Exploring the Impact of Dietary Sugar and Fasting on Cancer Risk and Prevention

**DOI:** 10.7759/cureus.67434

**Published:** 2024-08-21

**Authors:** Ashik Anil, Ronak Raheja, Diya Gibu, Aravind S Raj, S Spurthi

**Affiliations:** 1 Pharmacology and Therapeutics, East Point Hospital and Research Centre, Bangalore, IND; 2 Hematology and Medical Oncology, Manipal Hospitals, Bangalore, IND; 3 Biotechnology, SRM Institute of Science and Technology, Chennai, IND; 4 General Practice, Amrita Institute of Medical Science, Kochi, IND; 5 Immuno-Oncology Research, KLE University, Bangalore, IND

**Keywords:** cancer prevention, fasting, obesity and cancer, cancer cell metabolism, sugar and cancer

## Abstract

Over the last several years, the scientific community has grown concerned about the relationship between dietary sugar intake and cancer development. The main causes of concern are the increasing intake of processed foods rich in sugar and the rising incidence of cancer cases. This study aims to uncover the complex relationship between sugar consumption and cancer development and its progression, with a particular focus on investigating whether fasting can protect against this condition. Our review provides a detailed discussion of the molecular aspects of the sugar-cancer relationship and an analysis of the existing literature. It explains how sugar affects cell signaling, inflammation, and hormonal pathways associated with the development of cancer. We also explored the new role of fasting in the prevention of cancer and its impact on cancer patients. This encompasses fasting-triggered autophagy, metabolic alterations, and possible health benefits, which form the major concern of this paper. Thus, by deepening the knowledge of these relations and providing the results of the analysis accompanied by concise and meaningful illustrations to facilitate the understanding of the data, we open the door to the further development of ideas to minimize the rates of cancer and improve overall well-being.

## Introduction and background

In the current world of medical research, understanding the mechanism of cancer development has become the main focus. The increasing incidence of cancer in the world provokes scientists, practitioners, and policymakers to strive for further identification of its causes, risk factors, and approaches for its prevention and therapy. Cancer epidemiology points to a complex interaction between genetic, environmental, and lifestyle factors that influence the incidence and prevalence of cancer in populations [1]. Of these, dietary habits have come out as a focus of interest, especially concerning the intake of sugar. Nowadays, the search for sugar and cancer on the internet shows alarming warnings that sugar is a white poison and cancer's favorite food. The main reason behind this is the modern diet, especially the ubiquitous presence of sugar in modern diets, driven by a surge in processed foods and beverages, which make them high-calorie and high-energy dense, leading to an increased incidence of diet-related diseases, including certain types of cancer [2]. In a prospective study involving more than 70,000 participants without a history of cancer or diabetes who were surveyed using food frequency questionnaires, a follow-up over 7.2 years revealed 131 incident cases of pancreatic cancer (0.17% of the total population), leading to the conclusion that heightened consumption of sugar and high-sugar foods may correlate with an augmented risk of pancreatic cancer [3]. Positron emission tomography (PET) imaging, a crucial tool in oncology diagnostics, employs a radioactive substance such as fluorodeoxyglucose (FDG), which is structurally similar to glucose and is taken up by cells in a manner similar to glucose. They are used to visualize metabolic activity, particularly in cancerous cells, given their heightened metabolic rates. Monitoring changes in FDG uptake over time helps assess the efficacy of cancer treatments [4,5]. Despite the high sensitivity of PET scans, potential false results may occur due to factors like inflammation or specific cancers with low metabolic activity [6].

The raging debate over the potential link between excessive sugar consumption and cancer has sparked a paradigm shift in cancer prevention strategies. Fasting has emerged as a key focal point as the spotlight has shifted to innovative and holistic approaches. In recent years, scientific investigations into the impact of fasting on enhancing the response of cancer cells to chemotherapy and radiotherapy have yielded encouraging results [7]. This review article will, therefore, seek to provide a detailed analysis of the interplay between sugar intake and cancer, with a focus on the biological mechanisms that link the two. In this way, by providing a detailed description of the molecular and physiological effects of the potential benefits of fasting, we aimed to improve our knowledge of this important relationship and, thus, create a basis for developing effective prevention interventions for reducing the cancer burden in the modern world.

## Review

Sugar and cancer: an interplay

Sugar, commonly recognized as sucrose or table sugar, represents a carbohydrate that undergoes metabolic processes within the human body, yielding glucose, which serves as a basic and main energy source for the majority of cells [8]. Cancer cells typically exhibit rapid growth and multiplication rates compared to normal cells in the human body, necessitating a heightened demand for energy, primarily in the form of glucose [9]. The metabolic pathways of glucose differ between normal cells and cancer cells. Upon cellular uptake, glucose undergoes conversion into pyruvate, which serves as the terminal product of glycolysis and acts as a substrate for oxidative phosphorylation. Under aerobic conditions, pyruvate enters the mitochondria and is oxidized to acetyl CoA, which then combines with oxaloacetate to initiate the tricarboxylic acid (TCA) cycle and oxidative phosphorylation, which can generate 32 adenosine triphosphates (ATPs) [10]. Under anaerobic conditions, lactate dehydrogenase A (LDH-A) in the cytoplasm converts pyruvate to lactate, which is then exported into the extracellular space via monocarboxylate transporters (MCTs) [11]. 

**Figure 1:**
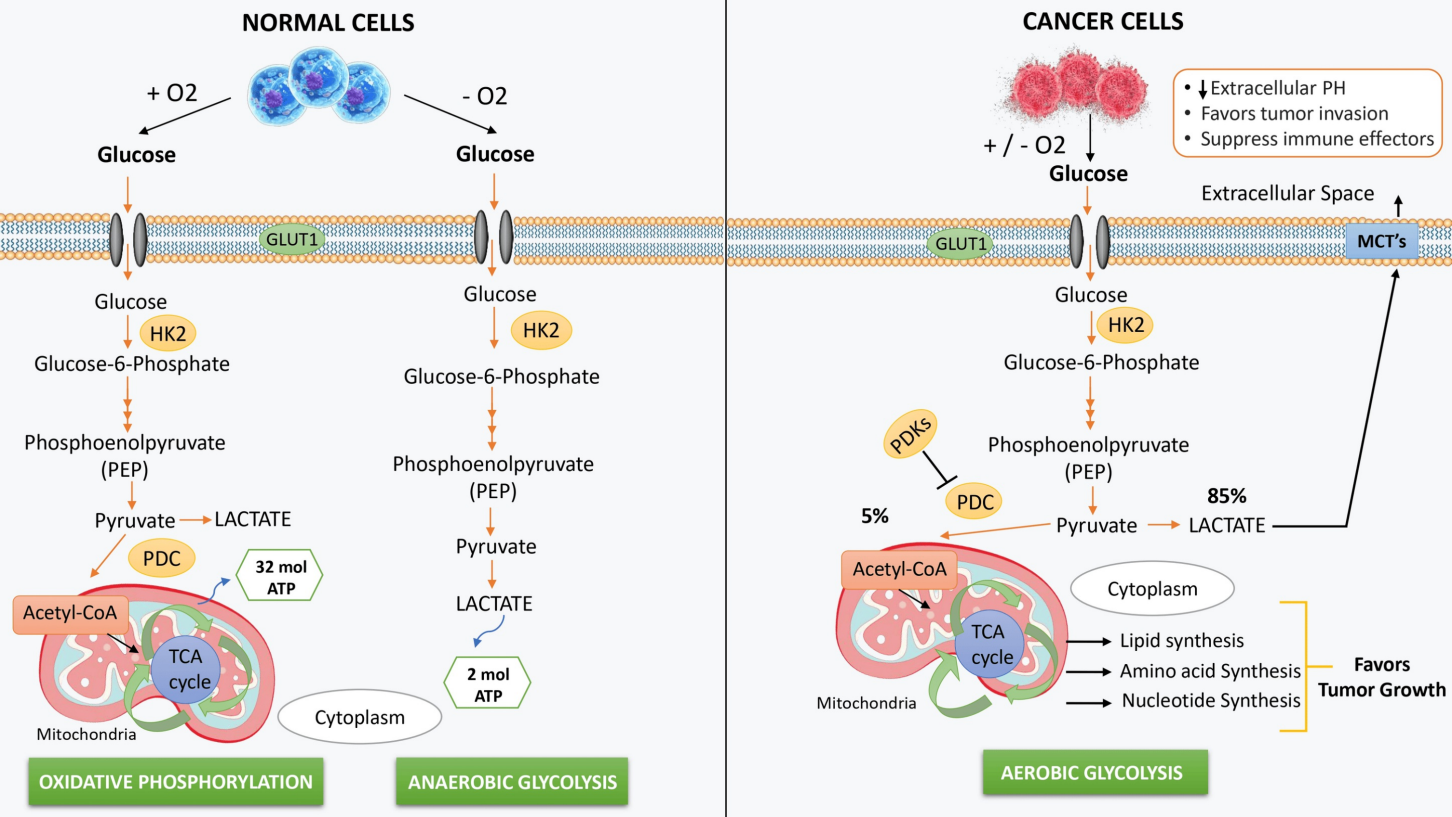
Glucose metabolism in normal cells and cancer cells based on Warburg theory O2: oxygen; HK2: hexokinase 2; PDC: pyruvate dehydrogenase complex; LDHA: lactate dehydrogenase A; PDK: pyruvate dehydrogenase kinases This figure is an original illustration created by the first author.

Over a century ago, in the 1920s, Otto Warburg made a significant discovery that tumors exhibit an unusually high demand for glucose compared to most healthy tissues [12]. Intriguingly, he found that the majority of glucose consumed by tumors follows a peculiar metabolic pathway, as in the cancer cells, the deliberate upregulation of pyruvate dehydrogenase kinases (PDKs) leads to the phosphorylation and subsequent inhibition of the pyruvate dehydrogenase complex (PDC). This alteration redirects pyruvate metabolism away from oxidative phosphorylation (OXPHOS) by impeding its conversion into acetyl-CoA. This distinctive metabolic behavior, known as 'aerobic glycolysis,' is noteworthy because it involves increased levels of fermentation even when oxygen is abundant, a departure from typical cellular responses to oxygen scarcity, where fermentation increases [13]. While aerobic glycolysis represents a fundamental element of metabolic processes across diverse organisms, its distinct association with cancer cells is commonly recognized as the 'Warburg effect' in the field of cancer biology. In cancer cells, glucose transporter 1 (GLUT1) expression is upregulated, resulting in an elevated demand for glucose [14]. Consequently, glycolysis is intensified, leading to increased pyruvate production and the subsequent accumulation of lactic acid. This accumulation contributes to a reduction in extracellular pH, creating a microenvironment conducive to tumor invasion while inhibiting immune responses [15]. Furthermore, the metabolic intermediates generated during aerobic glycolysis serve as essential building blocks for cellular component synthesis in rapidly dividing tumor cells, thereby promoting tumor growth [16]. The contrasting glucose metabolism in cancer and normal cells based on the Warburg effect is illustrated in Figure 1. 

Apart from glucose, fructose is an important energy source that stimulates the growth and spread of cancer cells. Fructose enters the cell via the glucose transporter GLUT5 and is phosphorylated by fructokinase via ATP depletion to generate fructose-1-phosphate [17]. These intermediates feed into the glycolytic pathway, thus bypassing the step within glycolysis, which is catalyzed by phosphofructokinase. The triose phosphates are transformed into glyceraldehyde-3-phosphate, and the glycolysis is further carried out to yield pyruvate and lactate in anaerobic conditions (Warburg effect). Further, these intermediates play a role in lipogenesis, providing for glycerol and fatty acid synthesis [18,19].

The increased ATP consumption during fructose phosphorylation will result in higher ADP and AMP accumulations. Elevated AMP levels are transformed into inosine monophosphate (IMP), which is then broken down by xanthine oxidase to create hypoxanthine, xanthine, and eventually uric acid. High uric acid levels cause oxidative stress since the body produces reactive oxygen species (ROS) which are created by high uric acid levels' reaction with mitochondrial DNA, proteins, and lipids, resulting in mitochondrial malfunction. As a result, ATP synthesis is reduced while increasing glycolysis and lipogenesis, even in the presence of oxygen [19-21]. Uric acid stimulates the NLRP3 inflammasome and enhances the secretion of pro-inflammatory cytokines, including IL-1β. Chronic inflammation can induce a tumor-supportive environment for cell replication, angiogenesis, and metastasis. High insulin levels caused by fructose-induced insulin resistance also raise IGF-1 levels, which foster cancer cell proliferation while suppressing apoptosis [22,23].

In recent years, the potential impact of glycative stress on cancer formation has garnered significant attention. This stress arises from the presence of exogenous or endogenous advanced glycation end products (AGEs) and highly reactive dicarbonyls [24]. The AGEs, formed through the interaction between reducing sugars and proteins, bind to and activate the receptor for advanced glycation end products (RAGE), a critical modulator of inflammation-associated cancer, and generate ROS that play a significant role in cancer development. Dicarbonyls, produced during glycolysis, lipid oxidation, or protein breakdown, include glyoxal, methylglyoxal, and 3-deoxyglucosone. These compounds not only contribute to the AGE pool but also induce carbonyl stress, leading to damage to carbohydrates, lipids, proteins, and DNA. This carbonylative damage can result in lesions that are implicated in the development of cancer, underscoring the importance of understanding glycative stress and its role in oncogenesis [25].

Multiple studies consistently establish a direct correlation between elevated sugar intake and an increased risk of cancer progression and invasion. A study discovered that exposure to fructose modified the cell surface glycan structure of human breast cancer MDA-MB 468 cells, resulting in enhanced migration and invasion and thus promoting a more aggressive phenotype [26]. Research indicates that overexpression of GLUT5 is linked to various cancers and is notably associated with chemotherapy resistance, particularly in colorectal cancer [27-29]. Consequently, GLUT5 has emerged as a significant therapeutic target in cancer treatment [30]. In a thorough investigation encompassing 10,713 middle-aged female participants in Spain, persistent consumption of sugar-sweetened beverages (SSB) was notably linked to a higher incidence of breast cancer among postmenopausal women [31]. Emerging evidence suggests that heightened sugar intake, particularly fructose, poses an elevated risk for the development of colorectal cancers, pancreatic cancers, and cancers related to adiposity [32-34]. 

These findings suggest that eliminating or limiting sugar from the diet can potentially impede cancer growth or prevent its development altogether. Some studies challenge the traditional Warburg effect in cancer cells due to their heterogeneity [35]. Not all cancer cells exhibit this effect equally, with some relying more on oxidative phosphorylation, especially in later stages. Recent research highlights metabolic flexibility in cancer cells, allowing them to switch between glycolysis and oxidative phosphorylation based on energy needs and nutrients. Therefore, their metabolic behavior is context-dependent [36]. While strict low-carb diets might negatively impact long-term health by cutting out essential fiber and vitamins, it is vital for cancer patients, who often face weight loss and stress, to maintain a nutritious diet. In contemporary diets, there is a noticeable surge in the intake of added sugars, contributing to increased obesity rates. This escalation in obesity is a significant factor in the rising incidence of chronic diseases such as type 2 diabetes, cardiovascular diseases, and various forms of cancer [37]. 

Link of sugar consumption with obesity and its role as a cancer risk factor

In recent decades, the Western diet has evolved significantly, with a large increase in the consumption of added sugars, primarily in the form of high-fructose corn syrup (HFCS) and sucrose. Sugar intake has increased due to a variety of factors, including the broad availability of sugary processed foods and beverages, the food industry's forceful marketing methods, and shifts in community dietary choices. High-fructose corn syrup has been a popular sweetening additive in a number of processed foods and fizzy beverages due to its inexpensive cost and ability to increase sweetness [38]. The prevalence of obesity has surged to epidemic levels in Western countries, concomitant with an escalating intake of sugar. It is widely acknowledged that obesity constitutes a significant risk factor for various malignancies, encompassing, but not limited to, breast, colorectal, endometrial, esophageal, and pancreatic cancers [39]. This association between obesity and cancer is multifaceted and complex, involving a range of mechanisms. Fat cells, also known as adipocytes, release inflammatory proteins known as adipokines, which are responsible for DNA damage and can eventually cause tumors [40]. When there are more fat cells, adipokine production and the production of pro-inflammatory cytokines like tumor necrosis factor-alpha (TNF-α) and interleukin-6 (IL-6) are also increased [41]. These cytokines have the capacity to initiate a persistent state of low-grade inflammation throughout the body, commonly referred to as adipose tissue inflammation. This sustained inflammatory condition is closely linked to DNA damage, as the inflammatory molecules can induce oxidative stress and disrupt normal cellular processes, potentially leading to mutations in the DNA [42]. 

The increase in adipose tissue is frequently associated with an upsurge in the generation of ROS, deleterious molecules formed as byproducts of cellular metabolism. Elevated ROS concentrations can induce oxidative stress, potentially leading to the occurrence of single- or double-strand breaks in DNA and hindering the intricate mechanisms responsible for DNA repair. This cascade of events ultimately culminates in the development of genetic mutations [43,44]. Furthermore, a relationship exists between obesity and metabolic dysfunction, which contributes to the development of insulin resistance. Obesity sets off a series of events that disrupt the normal pathways for insulin signaling, resulting in insulin resistance [45]. Obesity can lead to insulin resistance, a condition where the body's cells become less responsive to insulin. This resistance can cause elevated blood sugar levels, known as hyperglycemia. To counteract this, the pancreas produces more insulin to manage glucose levels, resulting in a condition called hyperinsulinemia [46]. Importantly, chronic hyperinsulinemia is associated with oxidative stress, a factor linked to the development of diverse types of cancer, including colorectal, liver, lung, and breast cancer [47,48]. Moreover, adipokines associated with obesity can impact hormonal signaling pathways. The human leptin gene, LEP, alternatively referred to as the "ob" gene, encodes a 16-kilodalton (kDa) protein composed of 167 amino acids. This protein was first isolated in 1994 by Jeffrey Friedman [49]. Leptin, recognized as the satiety hormone for its role in regulating body weight and energy balance, primarily operates within the hypothalamus. The association between obesity and disturbances in leptin signaling is well-established [50]. This disruption results in heightened cellular proliferation and the inhibition of programmed cell death (apoptosis), ultimately playing a contributory role in the onset of various cancer types [51]. 

**Figure 2:**
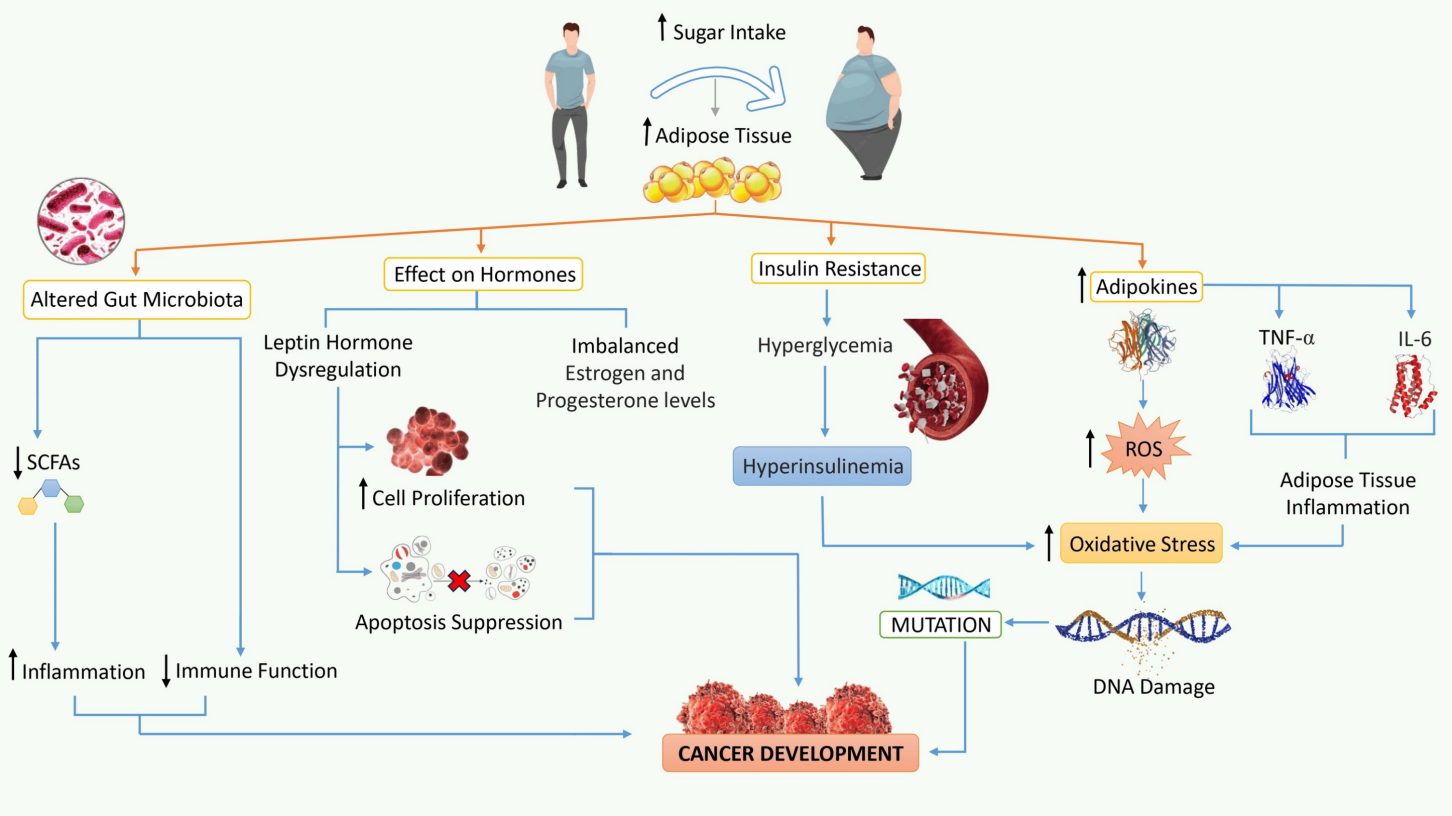
Pathophysiological relationship between obesity and cancer development SCFAs: short chain fatty acids; ROS: reactive oxygen species; TNF-α: tumor necrosis factor-alpha; IL-6: interleukin 6. This figure is an original illustration created by the first author.

Numerous studies have consistently shown that increased adipose tissue disrupts the body's hormonal balance, particularly estrogen and progesterone levels, with clear implications for cancer development [52]. A key mechanism behind this disruption involves pro-inflammatory cytokines produced by adipokines. These inflammatory molecules are directly associated with heightened estrogen synthesis, which, when excessive, can stimulate the proliferation of estrogen receptor-positive (ER+) breast cancer cells [53]. Also, accumulating evidence suggests that the hormonal imbalance between progesterone and estrogen, often linked to a higher body mass index (BMI), significantly causes mutations in the tumor suppressor genes and raises the risk of endometrial and breast cancers [54,55]. In addition, obesity caused by high-fat, high-sugar Western diets might alter the makeup and function of the gut microbiota [56]. This can cause beneficial bacteria in the gastrointestinal tract to produce fewer short-chain fatty acids (SCFAs) which are well-known for their anti-inflammatory effects and contribution to a healthy gut environment [57]. The alteration in these gut bacteria can lead to reduced SCFA production, potentially contributing to inflammation and an elevated risk of cancer. Additionally, the gut microbiota plays a pivotal role in modulating the immune system [58]. Changes in the gut microbiome can disrupt immune system function, diminishing its effectiveness in identifying and eliminating cancerous cells. The pathophysiological relationship between obesity and cancer development is illustrated in Figure 2. 

Fasting’s protective role against cancer

In the contemporary era, the prevalence of diets abundant in high-calorie and high-sugar fast food has contributed significantly to a spectrum of metabolic disorders, encompassing cardiovascular diseases, type 2 diabetes, and cancer. As a result, there is a burgeoning emphasis on uncovering effective methodologies to optimize our health. Intermittent fasting has emerged as a valuable strategy, not only for weight management but also for overall well-being. Diverging from traditional dietary advice that prescribes what to eat, intermittent fasting places emphasis on the timing of meals. While intermittent fasting has historical roots in religious and spiritual practices, its contemporary application predominantly centers around health enhancement and disease prevention [59]. It provides a versatile and adjustable approach to regulating calorie intake, offering a range of fasting schedules. Among the widely adopted intermittent fasting methods is the 16/8 strategy, wherein individuals refrain from eating for 16 hours daily and confine all meals to an eight-hour window. Scientific endorsement of intermittent fasting has been observed for its various health advantages, including but not limited to weight loss, improved cognitive function, potential anti-aging impacts, diminished insulin resistance, and the potential reversal of type 2 diabetes [60]. In this review article, our primary focus centers on elucidating the mechanisms underlying the role of intermittent fasting in preventing cancer.

Fasting provides various mechanisms that contribute to lowering the risk of cancer. A key mechanism involves the augmentation of autophagy, a crucial cellular process. Autophagy plays a pivotal role as cells dismantle and recycle impaired or dysfunctional components, such as proteins and organelles. This process is essential for sustaining a balanced cellular environment and fostering cellular survival [61]. During fasting, this process is bolstered by reducing the inhibition of autophagy initiation, primarily through the inhibition of a key regulator called the mammalian target of rapamycin (mTOR) which plays an important role in controlling cell growth and proliferation, particularly in cancer cells. By inhibiting mTOR, fasting removes a barrier to autophagy initiation, allowing for the formation of autophagosomes that capture and degrade cellular components [62]. Fasting also induces significant changes in the cellular energy state. As cellular ATP levels decline, an essential energy sensor called adenosine monophosphate-activated protein kinase (AMPK) becomes activated. This activation, in turn, triggers the activation of Unc-51-like autophagy-activating kinase 1 (ULK1), a critical initiator of the autophagy process [63].

Intermittent fasting assumes a pivotal role in cellular energy metabolism by instigating a transition in the body's predominant energy source, shifting from glucose to ketones and fatty acids. This metabolic shift is a consequence of reduced insulin levels during fasting, facilitating the utilization of stored fat for energy. As a result, adipose tissues are converted into ketones, serving as an alternative energy source, not only for the body but also for the optimal functioning of the brain [64]. This approach aids in diminishing the likelihood of cancer advancement as ketones prove to be less proficient energy sources for cancer cells. Moreover, intermittent fasting plays a pivotal role in weight management, thereby lowering the susceptibility to a spectrum of cancers. This is significant since obesity is linked to various mechanisms that can foster cancer initiation, as elucidated earlier. Additionally, intermittent fasting contributes positively to inflammation regulation and immune responses within the body. Empirical research has substantiated that fasting is effective in diminishing inflammatory markers like C-reactive protein (CRP) and IL-6 [65]. Moreover, the practice of fasting enhances the generation and functionality of immune cells, heightening their capacity to identify and eliminate proliferating cancer cells [66]. 

**Figure 3:**
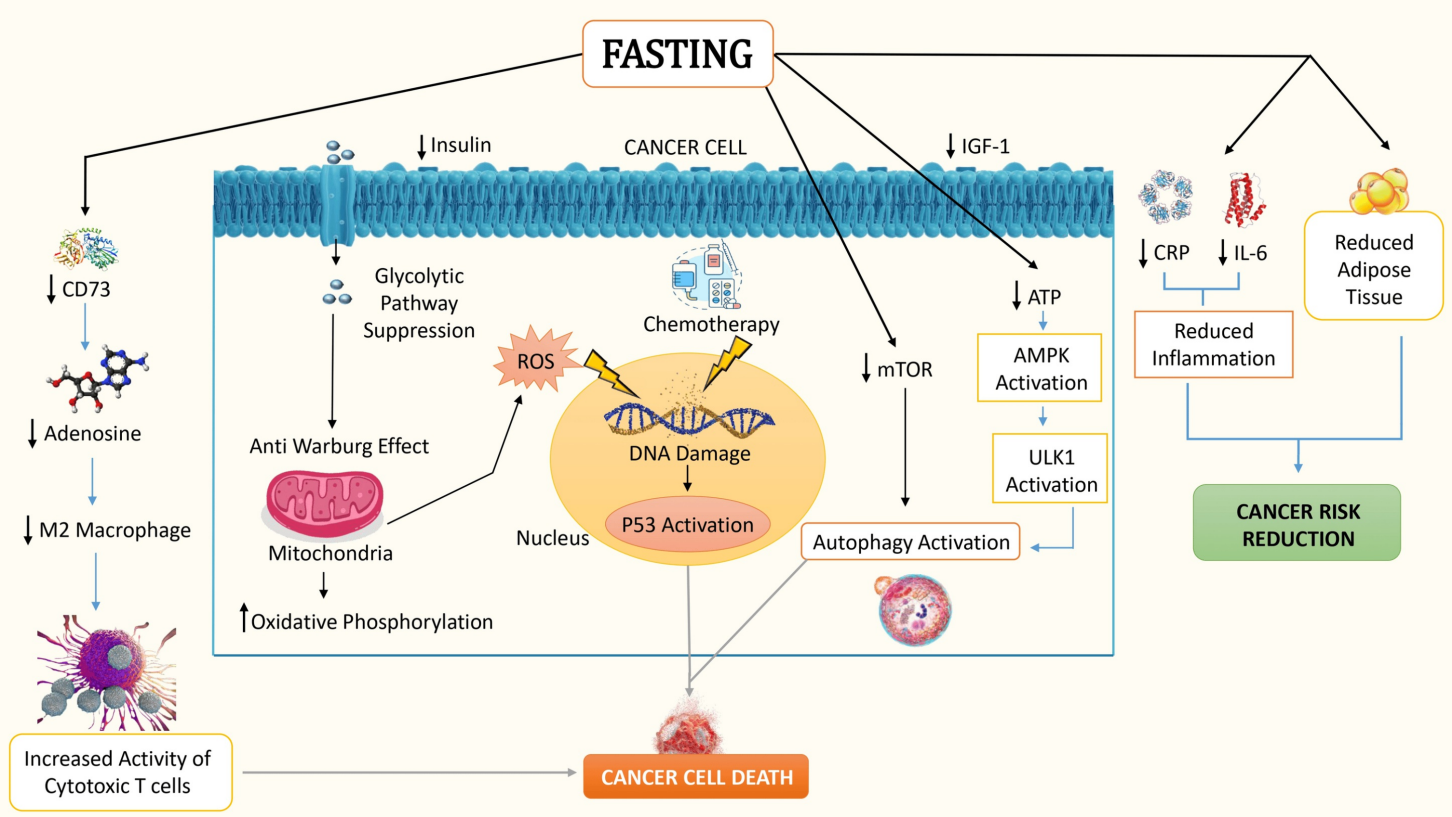
Mechanism of fasting in inducing cancer cell death and cancer risk reduction ROS: reactive oxygen species; ATP: adenosine triphosphate; AMPK: adenosine monophosphate-activated protein kinase; ULK1: unc-51-like autophagy-activating kinase 1; IL-6: interleukin 6; CRP: C-reactive protein; IGF-1: insulin-like growth factor 1. This figure is an original illustration created by the first author.

During periods of fasting, there is a decrease in the levels of IGF-1 and insulin, which are growth factors that support the growth and survival of cancer cells. In addition, fasting reduces glycolysis, causing an anti-Warburg effect that boosts the rate of oxidative phosphorylation within cancer cells' mitochondria [67]. The heightened oxidative phosphorylation observed in these cells results in an elevated generation of ROS, causing DNA damage. When coupled with the DNA damage induced by chemotherapy, this triggers the activation of P53, a tumor suppressor protein, ultimately culminating in the apoptosis of cancer cells [68]. Moreover, fasting can lead to a decrease in the expression of CD73 within cancer cells. CD73 is an enzyme responsible for generating adenosine, a molecule recognized for its immunosuppressive properties. The reduction in CD73 levels resulting from fasting subsequently diminishes adenosine production in the extracellular environment. As a result, this impedes the ability of immune cells, particularly macrophages, to adopt an immunosuppressive M2 phenotype [64]. Consequently, fasting contributes to the creation of a tumor microenvironment that is more conducive to immune activation than immune suppression. The role of fasting in cancer prevention and its involvement in inducing cancer cell death is illustrated in Figure 3.

Fasting has gained recognition not only for its potential role in cancer prevention but also as a supportive strategy for cancer patients undergoing chemotherapy, demonstrating both safety and benefits in numerous research investigations. We have organized some of the preclinical and clinical study data on these findings in Table 1.

**Table 1:** Clinical and preclinical studies highlighting the role of fasting and cancer

Author, year	Study type	Intervention	Key findings
Mindikoglu et al., 2020 [69]	Clinical	Intermittent fasting	Four weeks of intermittent fasting led to a significant increase in tumor suppressor and DNA repair gene protein products, along with a notable reduction in tumor promoter proteins.
Lee et al., 2012 [70]	Pre-clinical	Short-term fasting	In mouse neuroblastoma models, only the combination of fasting cycles and chemotherapy achieved long-term cancer-free survival.
Berrigan et al., 2002 [71]	Pre-clinical	Intermittent fasting	Mice with p53 deficiency showed reduced tumor-related mortality with calorie restriction and intermittent fasting.
Dorff et al., 2016 [72]	Clinical	Intermittent fasting	The preliminary evidence from the study suggests that fasting may offer protective benefits to normal tissues against platinum chemotherapy-induced damage.
Bias et al., 2017 [73]	Pre-clinical	Short-term fasting	The study demonstrated that short-term fasting effectively decreased the mortality rate of mice exposed to otherwise lethal doses of doxorubicin (DXR).
De Groot et al., 2015 [74]	Clinical	Short-term fasting	The study found that short-term fasting during chemotherapy for HER2-negative breast cancer patients reduced toxicity and aided DNA repair in white blood cells.

While fasting is usually accompanied by weight loss, its use in cancer patients must be carefully adapted to avoid unintended weight loss, which might worsen the outcomes. Fasting protocols, such as intermittent fasting or fasting-mimicking diets, should focus on improving metabolic health and reducing oxidative stress rather than promoting weight reduction. This diet should be carefully monitored by healthcare professionals to ensure that patients receive the right number of calories and all the essential nutrients they need. The aim is not to bring about dramatic weight reduction but rather to enhance cancer treatment modalities while, at the same time, preserving muscle mass and the overall nutritional status of the individual.

## Conclusions

In summary, our findings show a clear link between sugar consumption and cancer, with a particular focus on obesity as a major risk factor associated with increased sugar intake. It becomes clear that one of the key components of cancer prevention is maintaining a healthy body weight. Furthermore, by utilizing fasting's metabolic and cellular advantages, our analysis underscores the preventative benefits of fasting in reducing cancer risk. These insights provide helpful tactics for individuals who want to actively modify their lifestyles and reduce their risk of developing cancer.
